# Lymphatic leakage after pelvic lymphadenectomy for cervical cancer: a retrospective case-control study

**DOI:** 10.1186/s12885-021-08984-1

**Published:** 2021-11-18

**Authors:** Li Chen, Liang Lin, Ling Li, Zuolian Xie, Haixin He, Cuibo Lin, Jian Chen, An Lin

**Affiliations:** Department of Gynecology, Fujian Medical University Cancer Hospital, Fujian Cancer Hospital, Fuzhou, 350014 Fujian China

**Keywords:** Lymphatic leakage, Lymph node dissection, Cervical cancer

## Abstract

**Background:**

The study aims to evaluate the clinical features and management of postoperative lymphatic leakage (PLL) in patients with cervical cancer who received pelvic lymphadenectomy.

**Methods:**

This retrospective study screened consecutive patients with cervical cancer (stage Ia2-IIb).

**Results:**

Among 3427 cases screened, 63 patients (1.8%) were diagnosed with PLL, which manifested as persistent abdominal drainage (42/63, 66.7%), chylous ascites (12/63, 19.0%) or vaginal drainage (9/63, 14.3%). Median time from surgery to onset of PLL was 6 days (range, 4–21 days). All cases resolved in a median 10 days (range, 3–56 days) after conservative treatment; although one case experienced recurrence of vaginal drainage after 26 days, this also resolved after conservative therapy. Multivariate analysis showed that two cycles of neoadjuvant chemotherapy (odds ratio [OR], 3.283; 95% confidence interval [95%CI], 1.289–8.360; *P* = 0.013), a decrease in hemoglobin level of ≥20 and < 30 g/L (OR, 6.175; 95%CI, 1.033–10.919; *P* = 0.046) or ≥ 30 g/L (OR, 8.467; 95%CI, 1.248–17.426; *P* = 0.029), and postoperative albumin level ≥ 30 and < 35 g/L (OR, 2.552; 95%CI, 1.112–5.857; *P* = 0.027) or < 30 g/L (OR, 5.517; 95%CI, 2.047–18.148; *P* = 0.012) were associated with PLL.

**Conclusion:**

Neoadjuvant chemotherapy, postoperative anemia and postoperative hypoproteinemia are risk factors for PLL.

## Background

Postsurgical lymphatic leakage because of trauma to the lymphatic system is a known complication of abdominal surgery [1]. Various types of postoperative lymphatic leakage have been described including lymphatic ascites [2], lymphocele [3], lymphorrhea [4], lymphatic fistula [5], chylous ascites [6], chylorrhea [7], chyloretroperitoneum [8] and chylothorax [9]. An important cause of postoperative lymphatic leakage in patients with gynecological malignancies is pelvic and paraaortic lymphadenectomy [10]. The reported incidences of postoperative lymphatic ascites and chylous ascites in patients undergoing lymph node dissection for gynecological malignancies are 2.7–4.0% [10, 11] and 0.17–2.0% [12–14], respectively. In the aspect of abdominal surgery, a number of studies believe that the number of lymph node resection is closely related to the occurrence of chylous leakage [15, 16]. NACT may be a risk factor for chylous leakage, according to a study of chyle leakage after esophagectomy [17]. In addition, various risk factors for postoperative lymphatic leakage have been described. Cirrhosis and heart failure were considered to promote the occurrence of lymphatic leakage [18]. The extent of abdominal surgery, tumor grade, number of positive lymph nodes, number of lymph nodes harvested, neoadjuvant chemotherapy (NACT) were considered as risk factors of chylous leakage in relevant studies [16, 19, 20]. Early postoperative oral intake is considered to be a risk factor for chylous leakage in patients after pancreaticoduodenectomy [21], but this argument is still controversial [15].

The clinical features of postsurgical lymphatic leakage include abdominal distension, leakage of clear fluid per vagina, dyspnea, pain, nausea, vomiting, prolonged postoperative ileus, malnutrition and hypoproteinemia [1, 10, 11]. Computed tomography (CT), ultrasonography, magnetic resonance imaging (MRI), paracentesis, lymphangiography and lymphoscintigraphy can all facilitate the diagnosis of postoperative lymphatic leakage [1]. A variety of management techniques are available, such as conservative treatment, medium-chain triglycerides, total parenteral nutrition, somatostatin, drainage and surgery [1].

Since postoperative lymphatic leakage is uncommon, data are limited regarding the incidence of and risk factors for this complication after the surgical management of cervical cancer. Therefore, the aims of this retrospective study were to review the incidence, clinical features and management of lymphatic leakage in patients treated surgically for cervical cancer at our hospital during the past decade and to identify risk factors for the occurrence of this complication.

## Materials and methods

### Study design and study participants

This retrospective case-control study included consecutive patients diagnosed with lymphatic leakage after radical hysterectomy and pelvic lymph node dissection for cervical cancer at the Department of Gynecology, Fujian Cancer Hospital & Fujian Medical University Cancer Hospital between January 2006 and August 2017. The inclusion criteria were: 1) age 25–70 years; 2) cervical cancer followed 2009 FIGO staging criteria Ia2 to IIb; 3) treated using transabdominal/laparoscopic radical hysterectomy (type III) and pelvic lymph node dissection with/without bilateral salpingo-oophorectomy; and 4) a diagnosis of postoperative lymphatic leakage was made using the following criteria: i) continuous discharge of a clear, pale-yellow or chyle-like fluid from the abdominal drainage tube or vagina after surgery; ii) daily drainage volume > 200 mL; iii) drainage volume increased rapidly after feeding; iv) laboratory examinations of the drainage fluid revealed a positive chyle test, a total protein level about half that of plasma, similar electrolyte levels to plasma, and a triglycerides level > 110 mg/dL; and v) urinary fistula was excluded [13, 17]. The exclusion criteria were: 1) paraaortic lymph node dissection was also performed during surgery; 2) other surgical procedures were carried out during the operation, such as splenectomy, intestinal resection or intestinal neoplasty; 3) a second operation was performed after the initial surgery for any reason; 4) development of a urinary fistula or intestinal fistula after surgery; and 5) serious comorbid diseases, such as systemic lupus erythematosus or Sjögren’s syndrome, that required medical intervention or hormone therapy. In addition to the case group (i.e., patients diagnosed with postoperative lymphatic leakage), an equal number of patients without postoperative lymphatic leakage were enrolled as a control group. The patients in the control group were also selected from those who underwent radical hysterectomy and pelvic lymph node dissection for cervical cancer at our department. The control group of patients was matched 1:1 with the case group for age (within 5 years) and date of surgery (within 1 week); if more than one medical record met the requirements for matching, the medical record with the closest surgical time to the patient in the case group was selected. The pelvic systematic lymphadenectomy included the removal of internal iliac lymph nodes, external iliac lymph nodes, obturator lymph nodes, deep inguinal lymph nodes and common iliac lymph nodes. The extent of lymph node dissection was defined by the surface of iliopsoas muscle on the lateral side, the ureter on the medial side, 3 cm above the bifurcation of iliac artery as the upper boundary, deep inguinal lymph nodes where the deep circumflex iliac vein crosses the external iliac artery as the lower boundary, and obturator nerve plane at the bottom. All lymphoid adipose tissue should be completely removed in the above range. A drainage was routinely placed in all the patients during operation in order to observe the recovery of abdominal and pelvic wound after operation. If the related complications such as intra-abdominal hemorrhage, intra-abdominal infection, urinary fistula, intestinal fistula and lymphatic leakage are excluded, the drainage tube would be removed about 3–10 days after operation. If the corresponding complications appeared, the postoperative drainage should be retained until the complications were cured, the drainage fluid was clear, and the volume was less than 150 ml. For locally advanced patients, neoadjuvant chemotherapy or neoadjuvant radiotherapy or a combination of the two treatments were used. Neoadjuvant chemotherapy regimens mainly includes paclitaxel/carboplatin and paclitaxel/cisplatin commended by the guidelines. If the patient had a history of paclitaxel allergy, we would choose gemcitabine/platinum instead. The number of cycles was mainly determined by tumor regression. The dosage of intracavitary brachytherapy, as the neoadjuvant radiotherapy, was 10 to 20Gy which was completed within 2 weeks.

This study was approved by our hospital’s ethics committee, and the requirement for consent was waived because the analysis was retrospective. However, all patients provided informed consent for the treatments they received.

### Follow-up and collection of clinical data

All patients were followed-up monthly for the first 6 months, every 3 months from 6 months to 2 years, every 6 months from 2 years to 5 years, and annually thereafter. All patients included in the analysis were followed-up for at least 2 years. The following data were extracted from the medical records: age; height; weight; body mass index (BMI); FIGO (International Federation of Gynecology and Obstetrics) stage; histologic type of cervical cancer; comorbidities; number of NACT treatments; neoadjuvant radiotherapy use; type of surgery (laparoscopic or open); intraoperative blood loss; number of lymph nodes resected; presence/absence of lymph node metastasis; preoperative and postoperative levels of hemoglobin (measured using a colorimetric method) and albumin (measured using the bromocresol green method) in peripheral venous blood; and whether postoperative pelvic infection occurred. The postoperative levels of hemoglobin and albumin were measured at 2 days and 5 days after surgery, and the lowest value for each parameter were used in the analysis. Resolution of lymphatic leakage after treatment was defined as a drainage volume < 150 mL/day for more than 3 days, < 10 mL of vaginal drainage, or a reduction in the triglyceride level in the drainage fluid to < 110 mL/dL.

### Statistical analysis

The analysis was performed using SPSS 21.0 (IBM Corp., Armonk, NY, USA). All continuous measurement data were tested for normality and confirmed to have a normal distribution. Continuous data are presented as the mean ± standard deviation and were compared between groups using the t-test for independent samples. Categorical data are presented as *n* (%) and were compared between groups using the chi-squared test. Parameters that differed significantly (*P* < 0.05) between the case group and control group in the univariate analysis were entered into a multivariate conditional logistic regression analysis (using the enter method) to identify factors independently associated with postoperative lymphatic leakage (Table 1). Parameters were excluded from the multivariate analysis if they exhibited collinearity with other variables. Odds ratios (ORs) and 95% confidence intervals (95%CIs) were calculated. *P* < 0.05 was considered significant.

**Table 1:** Assignment of variables in the multivariate logistic regression analysis

	Value	Assignment in multivariate analysis
Preoperative albumin (g/L)	≥35	1
	< 35 and ≥ 30	2
	< 30	3
Postoperative hemoglobin (g/L)	≥110	1
	< 110 and ≥ 90	2
	< 90	3
Postoperative albumin(g/L)	≥35	1
	< 35 and ≥ 30	2
	< 30	3
Decrease in hemoglobin (g/L)	< 10	1
	< 20 and ≥ 10	2
	< 30 and ≥ 20	3
	≥30	4
Decrease in albumin (g/L)	< 5	1
	< 10 and ≥ 5	2
	< 15 and ≥ 10	3
	≥15	4
Number of lymph nodes resected	<25	1
	<35 and ≥ 25	2
	≥35	3

## Results

### Clinical characteristics of the study participants

Among 3427 patients with cervical cancer treated surgically at our hospital during the study period, 63 patients (1.8%) had lymphatic leakage after the operation. The clinical characteristics of these 63 patients with postoperative lymphatic leakage (case group) are shown in Table 2. The median time from surgery to development of lymphatic leakage was 6 days (range, 4–21 days). Postsurgical lymphatic leakage presented as persistent abdominal drainage of non-bloody fluid in 42 patients (average maximum daily drainage volume of 610 ± 127 mL), chylous ascites (confirmed by its milky color) in 12 patients, and vaginal discharge of fluid in 9 patients. Laparoscopic and open surgery were used in 24 patients (38.1%) and 39 patients (61.9%), respectively. The median number of lymph nodes resected was 32 (range, 18–62).

**Table 2:** Clinical characteristics of the study participants

Characteristic	Case group (***n*** = 63)	Control group (***n*** = 63)	***P*** value
Age	52.86 ± 8.56	51.59 ± 7.81	0.386
Height (cm)	157.78 ± 4.23	158.29 ± 4.17	0.499
Weight (kg)	58.25 ± 4.78	57.37 ± 5.86	0.352
Body mass index (kg/m^2^)	23.39 ± 1.67	22.85 ± 1.74	0.078
Comorbid diabetes mellitus			0.219
Yes	19 (30.2%)	13 (20.6%)	
No	44 (69.8%)	50 (79.4%)	
FIGO Stage			0.859
I	32 (50.8%)	31 (49.2%)	
II	31 (49.2%)	32 (50.8%)	
Histologic type			0.338
Squamous cell carcinoma	45 (71.4%)	38 (60.3%)	
Adenocarcinoma	12 (19.0%)	19 (30.2%)	
Other	6 (9.5%)	6 (9.5%)	
Number of NACT cycles			0.021*
0	26 (41.3%)	37 (58.7%)	
1	12 (19.0%)	13 (20.6%)	
2	25 (39.7%)	13 (20.6%)	
Neoadjuvant radiotherapy			0.051
Yes	37 (58.7%)	26 (41.3%)	
No	26 (41.3%)	37 (58.7%)	
Surgical method			0.672
Laparoscopy	24 (38.1%)	22 (34.9%)	
Laparotomy	39 (61.9%)	41 (65.1%)	
Duration of surgery (h)	3.2 ± 1.0	2.9 ± 0.9	0.983
Intraoperative bleeding volume (mL)	253 ± 75	256 ± 71	0.770
Number of lymph nodes resected	37 ± 7	34 ± 8	0.008*
Lymph node metastasis			0.052
Yes	6 (9.5%)	1 (1.6%)	
No	57 (90.5%)	62 (98.4%)	
Postoperative pelvic infection			0.045*
Yes	22 (34.9%)	12 (19.0%)	
No	41 (65.1%)	51 (81.0%)	
Preoperative hemoglobin level (g/L)	137.08 ± 4.15	136.52 ± 5.16	0.506
Preoperative albumin level (g/L)	39.68 ± 4.15	38.10 ± 5.16	0.000*
Postoperative hemoglobin level (g/L)	97.44 ± 8.44	101.40 ± 8.56	0.010*
Postoperative albumin level (g/L)	30.39 ± 2.14	31.40 ± 2.83	0.025*
Decrease in hemoglobin level (g/L)^§^	39.63 ± 8.83	35.13 ± 7.44	0.002*
Decrease in albumin level (g/L)^§^	9.29 ± 2.15	6.70 ± 2.34	0.000*

Data are presented as mean ± standard deviation or *n* (%). NACT: neoadjuvant chemotherapy. ^§^ Preoperative level minus postoperative level. * *P* < 0.05

The control group consisted of 63 patients who were matched with the case group for age and time of admission; the clinical characteristics of the control group are presented in Table 2. There were no differences between the case and control groups in age, height, weight, BMI, prevalence of comorbid diabetes mellitus, FIGO stage, histologic type of cervical cancer, use of neoadjuvant radiotherapy, preoperative hemoglobin level, surgical method, duration of surgery, intraoperative blood loss or prevalence of lymph node metastasis (Table 2). However, compared with the control group, the case group had significantly greater use of NACT, higher preoperative albumin level, greater number of lymph nodes resected, higher incidence of postoperative pelvic infection, lower postoperative levels of hemoglobin and albumin, and larger decreases in hemoglobin and albumin levels from before surgery to after surgery (all *P* < 0.05; see Table 2 for details).

### Management of postoperative lymphatic leakage

**Fig. 1:**
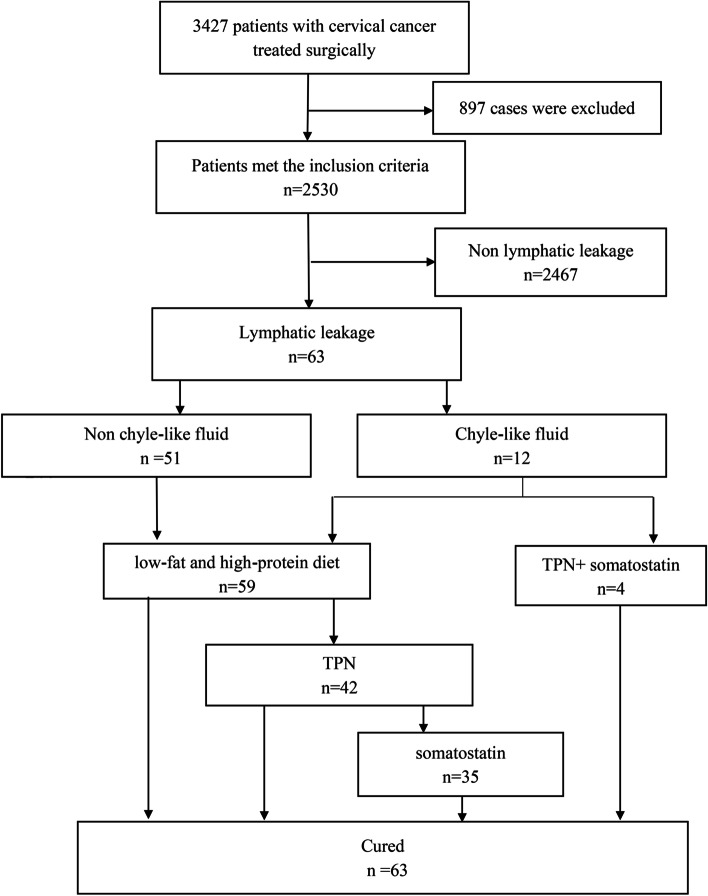
Flowchart of enrolment and analysis process

All cases with postsurgical lymphatic leakage resolved after conservative treatment (Fig. 1), which included routine placement of an indwelling drainage tube (*n* = 38), administration of a low-fat and high-protein diet (*n* = 59), total parenteral nutrition (*n* = 46) and somatostatin (*n* = 39). The median time from onset to resolution of lymphatic leakage was 10 days (range, 6–56 days). During the follow-up period, one case had a recurrence of vaginal fluid leakage 26 days after initially successful treatment; this resolved after 5 further days of conservative treatment and did not subsequently recur.

### Multivariate regression analysis of factors associated with postoperative lymphatic leakage

Based on the results of the univariate analyses, number of lymph nodes resected, NACT use, the decrease in hemoglobin level from before surgery and postoperative albumin level were entered into the multivariate regression analysis. Preoperative and postoperative hemoglobin levels, preoperative albumin level and decrease in the level of albumin to after surgery were not entered into the multivariate analysis due to collinearity with the decrease in hemoglobin level from before surgery and postoperative albumin level. Postoperative pelvic infection was also excluded from the analysis due to collinearity with albumin level (*p* = 0.031). The multivariate analysis showed that two cycles of NACT (OR, 3.283; 95%CI, 1.289–8.360; *P* = 0.013), a decrease in hemoglobin level of ≥20 and < 30 g/L (OR, 6.175; 95%CI, 1.033–10.919; *P* = 0.046) or ≥ 30 g/L (OR, 8.467; 95%CI, 1.248–17.426; *P* = 0.029), and postoperative albumin level ≥ 30 and < 35 g/L (OR, 2.552; 95%CI, 1.112–5.857; *P* = 0.027) or < 30 g/L (OR, 5.517; 95%CI, 2.047–18.148; *P* = 0.012) were significantly associated with postoperative lymphatic leakage (Table 3).

**Table 3:** Multivariate analysis of factors associated with postoperative lymphatic leakage

Factor		OR	95% CI	*P* value
Number of NACT cycles	0	–		
	1	2.679	0.876–8.197	0.084
	2	3.283	1.289–8.360	0.013*
Decrease in hemoglobin level (g/L)	< 10	–		
	≥10 and < 20	4.973	0.810–6.542	0.083
	≥20 and < 30	6.175	1.033–10.919	0.046*
	≥30	8.467	1.248–17.426	0.029*
Postoperative albumin level (g/L)	≥35	–		
	≥30 and < 35	2.552	1.112–5.857	0.027*
	< 30	5.517	2.047–18.148	0.012*
Number of lymph nodes resected	<25	–		
	<35 and ≥ 25	2.561	0.172–8.008	0.405
	≥35	3.049	0.221–12.011	0.081

NACT: neoadjuvant chemotherapy; OR: odds ratio; 95%CI: 95% confidence interval

*P < 0.05

## Discussion

This study found that only 63 of 3427 patients who underwent radical hysterectomy and pelvic lymph node dissection for cervical cancer were diagnosed with postoperative lymphatic leakage, corresponding to an incidence rate of 1.8%. Furthermore, the condition manifested as persistent abdominal drainage in 66.7% of cases, chylous ascites in 19.0% of cases, and vaginal drainage in 14.3% of cases. The time from surgery to onset of postoperative lymphatic leakage ranged from 4 to 21 days, and all cases resolved in a median 10 days (range, 3–56 days) after conservative treatment. Notably, two cycles of neoadjuvant chemotherapy, a decrease in hemoglobin level of ≥20 g/L after surgery and postoperative albumin level < 35 g/L were significantly associated with postoperative lymphatic leakage. Our findings show that postoperative lymphatic leakage is an uncommon complication of lymphadenectomy for cervical cancer and may be managed with conservative treatments. Since postoperative anemia and postoperative hypoproteinemia are risk factors for postoperative lymphatic leakage, attention should be made to meeting the nutritional needs of patients after surgery for cervical cancer.

Lymphatic leakage is an uncommon surgical complication. Prior studies of patients who underwent lymph node dissection for gynecological malignancies have reported incidences of postoperative lymphatic leakage varying from 0.17 to 4.0% [10–14]. The incidence of postoperative lymphatic leakage in the present study was 1.8%, which is comparable to that reported previously.

The occurrence of lymphatic leakage after surgery for cervical cancer likely results from damage to the lymphatic vessels. The extent of lymph node dissection is closely related to the occurrence of lymph leakage. In the present study, univariate analysis indicated that a significantly larger number of lymph nodes was dissected in the case group (patients diagnosed with postoperative lymphatic leakage) than in the control group. In agreement with our findings, other studies have also reported that a greater range of surgical dissection is associated with an increased risk of lymphatic leakage [15, 16, 18, 19]. However, there was no significant difference between the case group and control group in surgical method used (laparoscopic or open), in contrast to the findings of Perez-Medina et al. [20]. The characteristics of lymphatic leakage vary according to its location and the components of its lymphatic fluid. In particular, the fluid is clear or pale-yellow in color when lymphatic leakage occurs in the pelvic cavity but is milky when lymphatic vessels of the digestive tract are damaged due to the triglyceride-rich content.

An important finding of the present study was that although preoperative hemoglobin levels were similar between the case and control groups, a decrease in hemoglobin level of ≥20 g/L after surgery was independently associated with increased odds of lymphatic leakage. This is consistent with a previous univariate analysis that chylous ascites may be associated with the duration of surgery and intraoperative blood loss [21]. Although further research is needed to confirm our observations, we consider that the timely correction of postoperative anemia may decrease the risk of lymphatic leakage after surgery.

Our analysis also found that a lower postoperative albumin level was independently associated with lymphatic leakage. Furthermore, other studies have found that lower BMI may be a risk factor for lymphatic leakage [22, 23], implying that nutritional deficiency may enhance the risk of lymphatic leakage after an operation. We consider that the association between hypoproteinemia and postoperative lymphatic leakage may involve a decrease in the fluid pressure in the abdominal and pelvic cavities. Thus, we suggest that the provision of adequate nutrition to correct disturbances in plasma protein levels may reduce the risk of lymphatic leakage after surgery. Interestingly, the preoperative albumin level in the case group was slightly higher than that in the control group. We speculate that more attention may have been paid to perioperative nutrition (e.g., administration of protein supplements) in patients identified as having preoperative hypoproteinemia, which limited the decrease in their albumin levels after surgery. Although additional research is needed to fully characterize the relations of preoperative/postoperative albumin levels with lymphatic leakage, it is important that adequate perioperative nutrition be provided to all patients.

NACT and/or neoadjuvant radiotherapy can reduce the clinical stage of a tumor and make the tumor resectable. A notable finding of this study was that the use of two cycles of NACT was associated with increased odds of lymphatic leakage, in agreement with a previous report [22]. One possible reason for this is that the patients who received NACT would likely have had more advanced cancers and thus greater tumor invasion into local tissues, resulting in surgery that was more difficult and longer in duration, which in turn caused increased intraoperative damage to lymphatic vessels. Chemotherapy can also lead to hepatic dysfunction, immune dysfunction and poor nutritional status [24], leading to anemia and hypoproteinemia, and this may increase the risk of postoperative lymphatic fistula. However, patients undergoing neoadjuvant radiation (intracavitary brachytherapy) did not increase the risk of PLL. We speculate that the change of microenvironment in abdominal and pelvic cavity caused by this mode of radiotherapy is not enough to affect the formation of lymphatic leakage.

In the univariate analysis, postoperative pelvic infection was considered as a possible risk factor for lymphatic leakage after surgery. Postoperative pelvic infection may lead to changes in the micro-environment of the abdominal cavity, aggravating tissue edema and lowering the body protein level. However, collinearity with albumin level prevented this factor being entered into the multivariate analysis, so further research is needed to explore whether pelvic infection is related to lymphatic leakage.

Lymphatic leakage is usually managed conservatively, and the vast majority of cases resolve after the use of such therapies [18]. The available conservative treatments include routine placement of an indwelling drainage tube and provision of a low-fat diet supplemented with medium-chain triglycerides [25] with or without enteral nutrition or total parenteral nutrition. In addition, somatostatin has a good effect in patients with a large amount of transudate. Treatment should be individualized and adjusted to the severity of the lymphatic leakage and its consequences [26]. Although not all patients show recovery after fasting and somatostatin treatment, the initiation of a low-fat and high-protein diet for more than ten days usually achieves a curative effect through tissue self-repair. Lymphangiography is useful for detecting lymphatic leakage occurring after lymph node dissection, and lymphatic embolization is regarded as a new option in the treatment of lymphatic leakage [27, 28].

This study has some limitations. First, since this was a retrospective analysis, the results may be prone to selection bias and information bias. Second, the generalizability of the findings is not known because all patients were from a single institution. Third, the sample size was small, so the study may have been underpowered to detect some real differences between groups. Fourth, collinearity prevented the inclusion of some parameters in the multivariate analysis, so their possible effects on the risk of lymphatic leakage could not be evaluated. Fifth, other unknown confounding factors may have influenced the results. Multicenter, large-scale, prospective studies are needed to extend our observations.

In conclusion, postoperative lymphatic leakage is a rare complication of radical hysterectomy and pelvic node resection for cervical cancer. However, cases of postoperative lymphatic leakage can be managed successfully with conservative treatments. Since postoperative anemia and postoperative hypoproteinemia are risk factors for postsurgical lymphatic leakage, attention should be paid to actively correcting anemia and meeting the nutritional needs of patients after surgery for cervical cancer.

## Data Availability

Data are available upon reasonable request from corresponding author.

## Abbreviations

PLL: Postoperative lymphatic leakage
NACT: Neoadjuvant chemotherapy
CT: Computed tomography
